# A case series of intracranial dural arteriovenous fistulae mimicking cervical myelitis: a diagnosis not to be missed

**DOI:** 10.1007/s00415-021-10571-0

**Published:** 2021-04-26

**Authors:** Daniel Whittam, Saif Huda, Emily Gibbons, Richard Pullicino, Tom Solomon, Arun Chandran, Mani Puthuran, Anu Jacob

**Affiliations:** 1 Department of Neurology, The Walton Centre NHS Foundation Trust, Liverpool, UK; 2 Faculty of Health and Life Sciences, The University of Liverpool, Liverpool, UK; 3 Manchester Centre for Clinical Neurosciences, Salford Royal NHS Foundation Trust, Salford, UK; 4 Department of Interventional Neuroradiology, The Walton Centre NHS Foundation Trust, Liverpool, UK; 5 Department of Neurology, Cleveland Clinic Hospital, Abu Dhabi, United Arab Emirates

**Keywords:** Dural arteriovenous fistula, DAVF, Cognard type V, Myelitis, Myelopathy

## Abstract

**Objective:**

To describe the diagnostic features of intracranial dural arteriovenous fistulae (DAVF) presenting with cervical cord or brainstem swelling.

**Methods:**

Retrospective case note and neuroimaging review of patients with angiographically confirmed DAVF diagnosed during January 2015–June 2020 at a tertiary neuroscience centre (Walton Centre NHS Foundation Trust, Liverpool, UK).

**Results:**

Six intracranial DAVF causing cervical cord or brainstem oedema (all males aged 60–69 years) and 27 spinal DAVF (88% thoracolumbar) were detected over a 5.5-year period. Significantly more patients with intracranial DAVF received steroids for presumed inflammatory myelitis than those with spinal DAVF (5/6 vs 1/27, *p* = 0.0001, Fisher’s exact test). Several factors misled the treating clinicians: atypical rostral location of cord oedema (6/6); acute clinical deterioration (4/6); absence (3/6) or failure to recognise (3/6) subtle dilated perimedullary veins on MRI; intramedullary gadolinium enhancement (2/6); and elevated CSF protein (4/5). Acute deterioration followed steroid treatment in 4/5 patients. The following features may suggest DAVF rather than myelitis: older male patients (6/6), symptomatic progression over 4 or more weeks (6/6) and acellular CSF (5/5).

**Conclusion:**

Intracranial DAVF are uncommon but often misdiagnosed and treated as myelitis, which can cause life-threatening deterioration. Neurologists must recognise suggestive features and consider angiography, especially in older male patients. Dilated perimedullary veins are an important clue to underlying DAVF, but may be invisible or easily missed on routine MRI sequences.

## Introduction

Dural arteriovenous fistulae (DAVF) are acquired, direct connections between an artery and a vein without an intervening capillary network. Resultant venous hypertension may cause tissue oedema, hypoxia and ultimately, infarction. Spinal DAVF connect a radiculomedullary artery and vein, causing spinal cord oedema that classically manifests with an insidiously progressive or fluctuating myelopathy. Identification of dilated perimedullary veins through careful inspection of MR images is usually pivotal in making the diagnosis. Embolization or surgical disconnection can stabilize or improve neurological disability. However, late diagnosis is common, resulting in significant disability in many cases [1].

The vast majority of spinal DAVF are thoracolumbar; only 2% are cervical [2]. However, intracranial DAVF can also drain into spinal perimedullary veins as they enter the skull, causing brainstem or cervical cord oedema (Cognard type V DAVF) [3]. Such cases are considered rare, but are probably under-diagnosed and often mistaken for acute transverse myelitis (ATM), a far commoner cause of cervical myelopathy [4, 5]. Misdiagnosis may have grave consequences, because steroids, the standard treatment for ATM, can prompt rapid neurological deterioration in half of spinal DAVF cases [6]. Ascending cervical cord oedema could lead to ventilatory failure.

In this study, we identified all Cognard type V DAVF diagnosed at a tertiary UK neuroscience centre, which serves 3.5 million people. The objective was to review the diagnostic characteristics of these cases, identifying pitfalls and clues that may aid earlier differentiation from ATM.

## Methods

We searched our interventional neuroradiology database for angiographically confirmed DAVF between January 2015 and June 2020 (5.5 years). The case record and neuroimaging of each patient was retrospectively reviewed to identify clinical, radiographic and CSF characteristics. Written consent was obtained from all patients to publish their anonymised clinical data.

## Results

During a 5.5-year period, 33 DAVF were identified affecting the spinal cord. Twenty-seven were spinal DAVF (cervical [3], thoracic [16], lumbar [8]). Six were intracranial Cognard type V DAVF. The clinical, CSF, MRI and angiographic findings are summarised in Table 1. Steroids were administered more frequently for presumed ATM with Cognard type V DAVF (5/6) compared to spinal (1/27, cervical) DAVF (*p* = 0.0001, Fisher exact test).

**Table 1:** Summary of the clinical, CSF, MRI and angiographic findings for each case

	Case 1	Case 2	Case 3	Case 4	Case 5	Case 6
*Clinical features*						
Age (years)	60	65	62	64	69	63
Sex	Male	Male	Male	Male	Male	Male
Brief description of initial presentation	8 weeks of progressive walking difficulty and sphincter dysfunction, then sudden onset T10 paraplegia while defaecating	3 weeks of vomiting, then progressive paraparesis over 7 days	4 weeks of back pain and mild progressive paraparesis	8 months of mild walking difficulty, left upper limb weakness and frequent falls	4 weeks of mild urinary symptoms, followed by progressive mild (MRC grade 4/5) tetraparesis over 7 days	6 weeks of progressive lower limb sensory symptoms and sphincter dysfunction
Response to IV methylprednisolone 1000 mg daily for 3 days	Sensory level gradually ascended over next 3 days	–	Initial improvement, then 3 months later, rapid deterioration	Deteriorated over 2 days, such that he could no longer stand unaided	Rapid deterioration over 4 h to complete tetraplegia and respiratory failure	Transient mild worsening (increased lower limb stiffness and paraesthesia)
Approximate time from symptom onset to nadir (days)	61	28	126	250	38	42
Disability (mRS) at nadir	5	4	5	5	5	2
*CSF findings*						
White cell count (cells/mm^3^)	3 (N)	–	< 1 (N)	< 1 (N)	4 (N)	4 (N)
Protein (mg/dL)	64 (H)	–	30 (N)	120 (H)	69 (H)	89 (H)
Oligoclonal bands	Negative	–	Negative	Negative	Positive	Negative
IgG index	0.48 (N)	–	0.44 (N)	–	0.50 (N)	0.40 (N)
*MRI findings*						
T2-hyperintensity: longitudinal position	CMJ–T5	Pons–medulla	C2–C7	C3–T3	PMJ–T1	C1–C5
T2-hyperintensity: axial position	Central	Asymmetric	Central	Central	Central	Central
Cord expansion	Yes	Yes	Yes	Yes	Yes	Yes
Gadolinium enhancement	No	No	No	Yes (subtle)	No	Yes (marked)
Cord surface flow voids	No	Yes, but only on Gad-enhanced sequence, missed by referring team	Yes, but very faint and initially missed*	No	Yes, but very faint and initially misinterpreted**	No
*Angiographic findings and outcomes*						
DAVF feeding vessel	Ascending pharyngeal	Ascending pharyngeal & middle meningeal	Ascending pharyngeal	Occipital meningeal	Anterior inferior cerebellar	Medial tentorial
Definitive treatment	Embolization	Embolization	Surgical disconnection	Embolization	Embolization	Surgical disconnection
Time from first MRI to DSA (days)	28	10	91	33	7	78
Time from symptom onset to DSA (months)	2.8	1.2	3.9	9.1	1.4	3.9
Outcome (mRS) at 6 months post-treatment	4	2	4	2	1	1
Mobility at 6 months post-treatment	Wheelchair	Unaided, max. 1 mile	Wheelchair	Bilateral support, max. 30 yards	Unaided, unlimited	Unaided, unlimited

*Flow voids were visible on the initial MRI of case 3 but were missed at his first presentation and only identified when he re-presented 3 months later

** Flow voids were visible on the initial MRI of case 5 but were falsely attributed to his previous history of an intramedullary cavernoma at C7/T1 treated surgically 9 years prior

Abbreviations: IV, intravenous; mRS, modified Rankin score; N, normal; H, high; CMJ, cervicomedullary junction; PMJ, pontomedullary junction; DSA, digital subtraction angiogram

### Clinical findings

All Cognard type V DAVF patients were males in their 7th decade with a symptom duration of 1–8 months. Acute or hyperacute deterioration was observed in 4/6 cases. Clinical deterioration after steroid treatment occurred in 4/5 cases. Case 5 developed bulbar and respiratory failure 4 h after receiving steroids. Case 3 initially improved following steroids but deteriorated acutely 3 months later.

### MRI and CSF findings

**Fig. 1:**
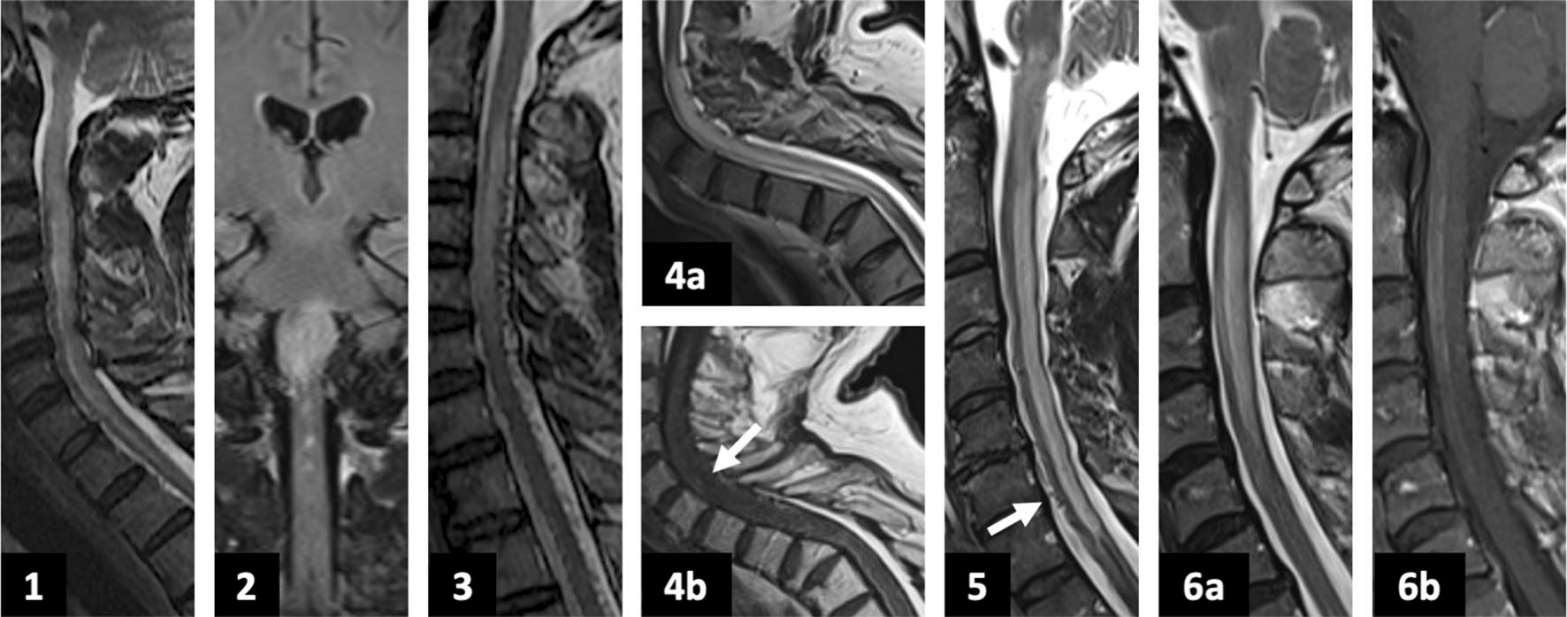
MRI images at presentation in each of the cases. (1) Case 1: sagittal T2-weighted image showing cervical cord swelling and signal abnormality from the cervicomedullary junction to C5. (2) Case 2: coronal T2-FLAIR image showing signal abnormality in the pons and medulla. (3) Case 3: sagittal T2-weighted image showing intramedullary signal abnormality at C2–C7 with cord expansion. Numerous flow voids are visible on the dorsal surface of the cord, indicating dilated perimedullary vessels. (4a) Case 4: sagittal T2-weighted image showing intramedullary signal abnormality at C3–T3 with cord expansion. (4b) Case 4: sagittal gadolinium-enhanced T1-weighted sequence showing subtle pathological gadolinium enhancement in the anterior cord (white arrow). (5) Case 5: sagittal T2-weighted image showing intramedullary signal abnormality from the pontomedullary junction to T1. There are some subtle flow voids on the cord surface (white arrow), but these were initially felt to relate to the patient’s history of an intramedullary cavernoma at C7/T1 surgically treated 9 years prior. (6a) Case 6: sagittal T2-weighted image showing intramedullary signal abnormality from C1–C5 with cord expansion. (6b) Case 6: sagittal gadolinium-enhanced T1-weighted sequence showing pathological gadolinium enhancement in the anterior cervical cord at C1–C5

**Fig. 2:**
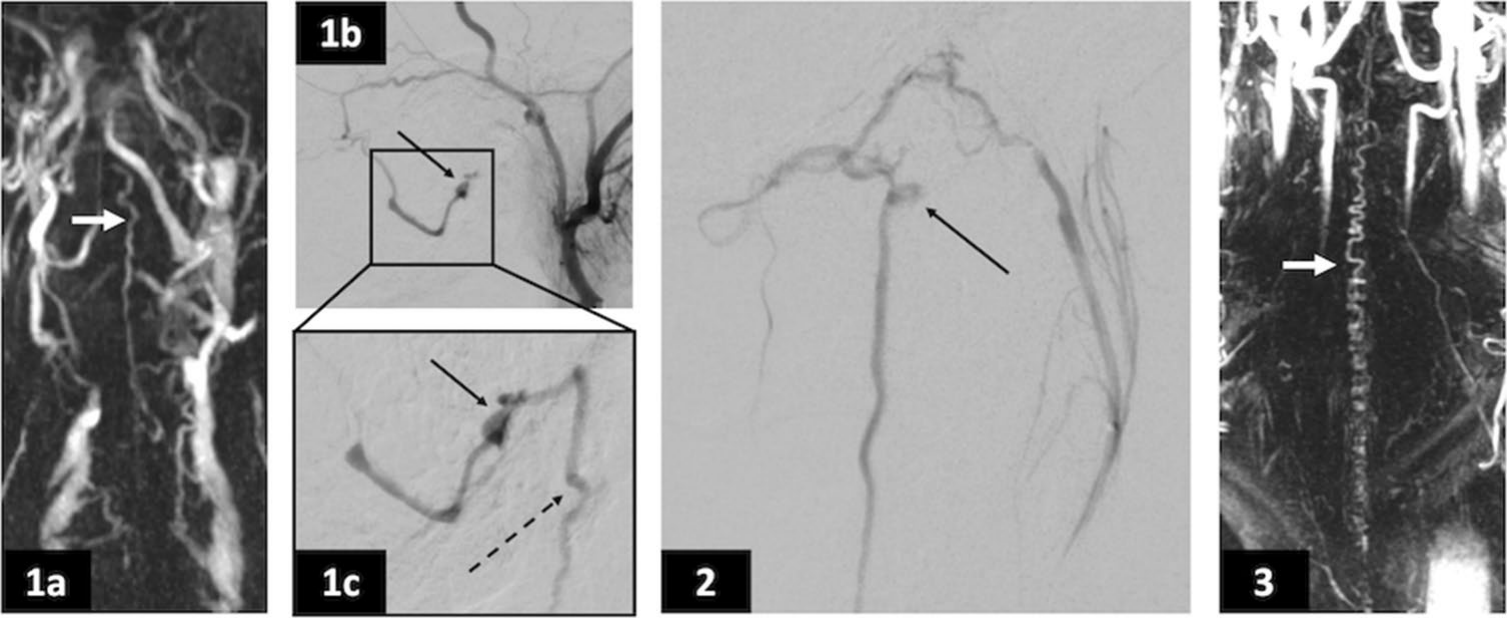
A selection of angiography images from cases 1–3. (1a) Case 1: MR angiogram (MRA) of the neck in the antero-posterior view showing a dilated vessel on the cord surface (white arrow), which represents early filling of the anterior spinal vein. (1b) Case 1: arterial phase digital subtraction angiogram (DSA) showing contrast injection of the right internal carotid artery. The fistula (black) arrow is on the skull base close the hypoglossal canal and is fed by the hypoglossal branch of the ascending pharyngeal artery. (1c) Case 1: zoomed in view of Fig. 1b, now in the late-arterial phase, showing the fistula (solid arrow) filling the anterior spinal vein (dashed arrow). (2) Case 2: late arterial phase DSA showing contrast injection of the left ascending pharyngeal artery. The fistula (black arrow) is shown draining into the anterior spinal vein. (3) Case 3: MRA of the neck in the antero-posterior view showing a tortuous vessel on the cord surface (white arrow), which represents early filling of the anterior spinal vein

Figure 1 shows the initial MRI of each case. Figure 2 comprises a selection of angiography images from cases 1–3. Five cases demonstrated longitudinally extensive intramedullary T2-hyperintensity with cord expansion. One patient had isolated brainstem oedema (Fig. 1.2). Intramedullary gadolinium enhancement was present in 2/6 cases (Fig. 1.4b, 6b). Dilated perimedullary veins were visible in 3/6 cases but were recognised only on specialist neuroradiology review. Perimedullary veins were visible as flow voids on T2-weighted sequences in two cases (Fig. 1.3, 5) but only on the gadolinium-enhanced T1-sequence of case 2 (not shown). CSF was obtained in 5 cases; white cell count was normal but 4/5 had elevated CSF protein levels (range 30–120 mg/dL, reference range 15–40 mg/dL).

### Outcomes

Four DAVF were amenable to microcatheter-directed embolization with Onyx liquid embolic agent and two required surgical disconnection via craniotomy. All patients gained some functional recovery post-treatment. Table 1 shows modified Rankin scores pre- (median 5, range 2–5) and 6 months post-treatment (median 2, range 1–4). At last follow-up, three patients remain independently mobile, one walks with bilateral support, one is a wheelchair user and one patient (case 1), also a wheelchair user, has died 3.6 years post-embolization from soft tissue infection and sepsis.

## Discussion

Over a 5.5-year period, only six Cognard type V DAVF were identified, but this equated to 1 for every 4.5 cases of ‘typical’ spinal DAVF. Inevitably, due to the difficulty of diagnosing DAVF, an unknown number of cases could remain undetected, even retrospectively. Frequent misdiagnosis (83%) and potentially life-threatening consequences of steroid administration were observed. Only patients with cervical cord swelling received steroids. This is presumably because it is usually lower thoracic cord swelling that leads neurologists to consider the possibility of DAVF. Cervical myelopathies are most often inflammatory, including multiple sclerosis (MS) and neuromyelitis optica spectrum disorders (NMOSD). Clinicians were probably also misled here by ‘inflammatory’ CSF abnormalities and imaging pitfalls, including intramedullary gadolinium enhancement and the absence of dilated perimedullary veins.

Demographic characteristics appeared important clues to an underlying DAVF. Our series aligns with the previously reported preponderance for spinal DAVF to affect older males. Five males for every one female are diagnosed with spinal DAVF and only 1% present before age 30 years [2]. Conversely ATM occurs frequently in young patients, and aquaporin-4 antibody-mediated NMOSD, the archetypal cause of longitudinally extensive ATM, preferentially affects females (sex ratio 3:1–9:1) [7]. Thus, DAVF should be strongly considered in all older male patients with longitudinally extensive cord swelling, both cervical and thoracolumbar.

Temporal progression is also important to consider. At presentation, all cases had been symptomatic for over 4 weeks, whereas ATM reaches a nadir within 3 weeks [8]. Four patients experienced an acute on chronic deterioration, which mimicked ATM, but this is recognised to occur with DAVF, particularly with surges in venous pressure (exercise or Valsalva manoeuvre). In a series of spinal DAVF, 63% progressed gradually, 26% had episodes of acute deterioration superimposed on a gradually progressive course, and 5% experienced an acute onset [9]. Progression of symptoms beyond 4 weeks should, therefore, be considered a red flag suggestive of a non-ATM diagnosis, and acute deterioration should not stop clinicians looking for a DAVF.

Importantly, steroids can cause dramatic clinical deterioration of DAVF, probably through transient mineralocorticoid-induced hypervolemia and venous hypertension [4–6, 10]. This can be life-threatening with high cervical cord involvement, as observed in our case of sudden respiratory failure. A case of transient clinical improvement was also identified. This phenomenon has been reported previously and may reflect reduction of cord oedema [11, 12].

CSF findings in DAVF are poorly studied, but albuminocytological dissociation was reported as early as 1926 by Charles Foix and Théophile Alajouanine (likely the earliest description of spinal DAVF) [13] and in contemporary angiographically confirmed cases [10]. In this series, 80% demonstrated albuminocytological dissociation. Elevated CSF protein is a non-specific finding that does not always indicate inflammatory or infective pathology. Mild elevation may be seen purely as an age-related phenomenon in those over 50 years, and vascular disorders, including DAVF, can cause CSF protein elevation through blood-CSF barrier disruption. In the context of longitudinally extensive cord swelling, the CSF white cell count is more reliable marker of inflammation. Acellular CSF is unusual in the context of longitudinally extensive ATM and is, therefore, an important red flag to look for a vascular aetiology.

The diagnosis of DAVF usually rests on standard MRI, can be guided by MR angiography (MRA) and is confirmed by digital subtraction angiography (DSA). In T2-weighted MRI, DAVF-related cord oedema is hyperintense, longitudinally extensive, centromedullary and poorly delineated [14]. Cognard type V DAVF have been reported to cause a characteristic pattern of central brainstem oedema with sparing of the periphery as well as internal linear segments in a tigroid pattern [15]. Asymmetric brainstem involvement, as seen in case 2, has also been reported [12].

Cord surface flow voids, caused by dilated perimedullary veins, strongly suggest a DAVF and must be carefully looked for. Specialist neuroradiologist review improved detection in our series, but flow voids were nevertheless identified in only 33%. They may be absent if the shunt volume is small, and conversely, pulsation artefact or venous obstruction by gross cord swelling may mimic DAVF [14]. A review of published Cognard type V DAVF cases reported a 37% sensitivity of T2-weighted MRI for spinal vein enlargement [16], which is similar to its sensitivity for spinal DAVF [17]. Gadolinium improves sensitivity for enlarged perimedullary veins, as seen in case 2. In one study, careful examination of Gadolinium-enhanced T1-weighted images improved detection of dilated perimedullary vessels from 37 to 76%, demonstrating a clear benefit of contrast administration for Cognard type V DAVF diagnosis [12].

Intramedullary gadolinium enhancement was observed in 33% of our cases, which is frequently considered indicative of inflammatory, infectious or neoplastic lesions. However, pathological enhancement is reported in half of spinal DAVF cases, due to blood-spinal cord barrier disruption [14, 17]. A non-enhancing cord segment amidst the enhancement (‘the missing piece sign’) due to inconsistent venous drainage, may be particularly suggestive of spinal DAVF over ATM [18]. Cognard type V DAVF have been reported to cause intense central or patchy brainstem enhancement, which may be mistaken for brainstem glioma [12, 15].

First-pass gadolinium-enhanced MRA can further aid diagnosis, by demonstrating early venous filling and helping to localise the shunt, which may be remote from the cord oedema [14]. This can avoid injections of all possible arterial feeders during DSA, which remains the gold standard diagnostic test for DAVF. Critically, investigation of suggestive cervical cord lesions must include cranial angiography, including selective injection of the external carotid arteries.

All patients in this series improved following obliteration of their DAVF but had residual disability. As a treatable condition, early diagnosis minimises long-term sequelae. Unfortunately, diagnostic delays are common, even with typical thoracolumbar DAVF. In one series, median diagnostic delay was 6 months, during which 96% of patients deteriorated [1]. The stakes are even higher with cervical cord involvement, underscoring the importance of considering DAVF in this context.

## Conclusion

Although uncommon, intracranial DAVF may mimic cervical myelitis, and should be considered in all cases of longitudinally extensive cord swelling. An integrated diagnostic approach incorporating clinical, CSF and MRI findings can identify supportive features. Specialist neuroradiology opinion, Gadolinium-enhanced MRI and early angiography could prevent serious harm from diagnostic delay or steroid administration.

## Data Availability

Participant data are stored securely at The Walton Centre NHS Foundation Trust.
